# Molecular feature of neutrophils in immune microenvironment of muscle atrophy

**DOI:** 10.1111/jcmm.17495

**Published:** 2022-07-27

**Authors:** Zongqi You, Xinying Huang, Yaoxian Xiang, Junxi Dai, Junjian Jiang, Jianguang Xu

**Affiliations:** ^1^ Department of Hand Surgery, Huashan Hospital Fudan University Shanghai China; ^2^ Key Laboratory of Hand Reconstruction, Ministry of Health Shanghai China; ^3^ Shanghai Key Laboratory of Peripheral Nerve and Microsurgery Shanghai China; ^4^ School of Rehabilitation Science Shanghai University of Traditional Chinese Medicine Shanghai China

**Keywords:** atrophy, immune microenvironment, Neutrophils, skeletal muscle

## Abstract

Homeostasis in skeletal muscle is sustained by the balance of functional and physical interactions between muscle and myofibre microenvironment. Various factors, such as ageing, disuse and denervation, tip the balance and induce skeletal muscle atrophy. Skeletal muscle atrophy, which involves complex physiological and biochemical changes, is accompanied by adverse outcomes and even increased mortality. Multiple studies have investigated the role of neutrophils in atrophied skeletal muscles; however, neutrophil intrusion in muscle is still a polemical knot. As technical obstacles have been overcome, people have gradually discovered new functions of neutrophils. The classical view of neutrophils is no longer applicable to their biological characteristics. To date, no clear association between the hidden injurious effect of neutrophil intrusion and muscle atrophy has been convincingly proven. Throughout this review, we have discussed the neutrophil activities that mediate muscle atrophy for distinct disease occurrences. Hopefully, this review will help both clinicians and researchers of skeletal muscle atrophy with relevant targets to further explore efficient medical interventions and treatments.

## 1 INTRODUCTION

Skeletal muscle is a particular structure that induces functional and structural alternation in answer to amounts of activity and mechanical load, and it carries out the vital work of strength development.^1^ As a rule, muscle atrophy is readily triggered by load decline. For instance, microgravity exposure, plaster fixing for treating a fracture, hind limb suspension of the rat and long‐term lying down cause static atrophy.^2^, ^3^, ^4^ With diseases or ageing of the nervous system, muscle atrophy can be observed as well.^5^ Regrettably, our understanding of this process remains unclear, and it is vital to understand the molecular mechanisms of muscle atrophy from the viewpoint of muscular atrophy prevention.

Skeletal muscle atrophy, irrespective of the original cause, results in typical cascades of events regulated by cells from the immune system.^6^ Therefore, a particularly important and unstudied aspect of muscle atrophy is the role of immune cells in aggravating damage and probably guiding muscle repair.^7^ Pathologic injury recruits cells (polymorphonuclear leukocytes and monocytes/macrophages) from the innate immune system that primarily clear cellular debris and also release noxious molecules.^8^ Moreover, infiltrating immune cells remain alive and are continuously activated, inducing tissue inflammation.^9^ The inflammatory reaction may generate some injury in situ and result in fat accumulation and deposition of collagen with interstitial fibrosis, despite representing a reaction to injury and necrosis.^10^ Similar events probably occur during consistent muscle injury which progresses to atrophy. Over the past few years, researchers have started to pay attention to the complex crosstalk between skeletal muscle and neutrophils in acute injury or chronic diseases.^9^, ^11^, ^12^

Neutrophils are the most abundant leukocytes in circulation, depending on steady supply from the bone marrow.^13^ Furthermore, they are the shortest‐living cells in mammals and are terminally differentiated with limited transcriptional activity. Under physiological conditions, they are the immune system's first line of defence against fungal and bacterial infection and employ rich antimicrobial elements (containing hydrolytic enzymes and reactive oxygen species [ROS]), capable of combating invasive pathogens. Additionally, neutrophils play a crucial part in the coordination of the inflammatory response and overall immunity. The diversity of their individual activities and the way they engage in biological processes (such as neutrophil extracellular traps [NETs]: the extrusion of their genomic DNA), render neutrophils unique.^14^ Neutrophils not only lead to tissue injury in inflammatory diseases and various autoimmune diseases but also play significant roles in disease development.^15^ Therefore, the neutrophils seem to be exciting targets for therapeutic intervention; nevertheless, we need to distinguish conducive responses from potentially detrimental side effects despite their crucial but complicated involvement in diverse diseases.

The abnormal chemotaxis of neutrophils in response to changes in environmental homeostasis may result in widespread cell injury. Notably, the mechanistic connections and interplay between skeletal muscle atrophy and neutrophils have raised considerable research interest. Multiple studies demonstrate that neutrophils and their migratory infiltration may cause predominant signalling events in skeletal muscle atrophy.^16^, ^17^ Therefore, elucidating the relationship between skeletal muscle atrophy and neutrophils is also a critical key to exploring therapeutic intervention for potential biological targets in skeletal muscle atrophy.

In this review, we provide a comprehensive summarization of neutrophil biology and discuss the established pathogenic roles of neutrophils in muscle atrophy. Distinct challenges and limitations, specific therapeutic strategies, and integral conceptual framework faced in targeting neutrophils are then evaluated. These findings enable us to distinguish between inflammatory processes that disrupt muscle homeostasis and the processes that promote muscle repair and regeneration during muscle atrophy. Additionally, we discuss the most recent studies describing muscle‐derived mediators which may promote or suppress the invasion of neutrophils. So far, investigations have revealed that neutrophils promote muscle injury after infiltration; however, the mechanism of neutrophils' role in muscle repairment has not been elucidated yet. Limiting certain aspects of inflammation may theoretically ameliorate and decrease signals for muscle atrophy. Considering muscle atrophy to be the result of immune cells competing with different components of the muscular microenvironment helps explain what makes neutrophils fascinating as they may be indispensable to the process of muscle atrophy.

## 2 OVERVIEW OF NEUTROPHILS

Neutrophils are evolutionarily old cells that acquired the tendency to phagocytize targets.^18^ These phagocytizing cells are present all over the faunal kingdom from invertebrates to mammals. In humans, neutrophils compose approximately 60%–70% of the total leukocytes and are the most abundant innate immune cells in the peripheral blood.^19^ While they are generated in the bone marrow, they spend limited time in circulation and are considered short‐lived cells. Generally, neutrophils have a transient circulating half‐life of about 6–8 h in mice and humans.^13^ Nevertheless, they perform diverse roles in inflammatory and immune processes.

**FIGURE 1:**
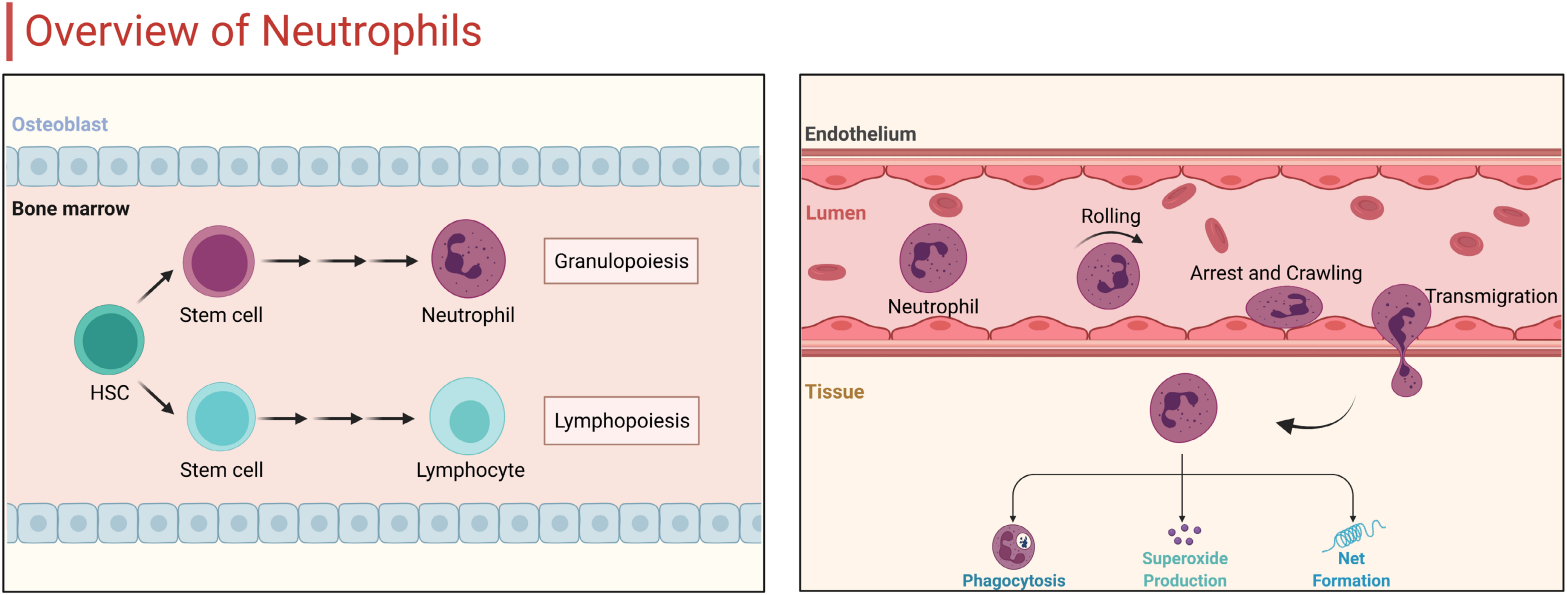
Overview of neutrophil development and function. Initially, neutrophils occur generation and maturation in the bone marrow. On sensing environmental microbial or inflammatory stimuli, neutrophils undergo a special recruitment cascade including tethering, rolling, arrest, crawling and eventually transmigrate into the target tissues. At the site of tissues, neutrophils exert their different effector functions, mainly consisted of phagocytosis, superoxide production and net formation.

The classical view of neutrophils is that they are the first line of cellular defence against invading microorganisms by rapidly crossing the blood endothelial cell barrier and performing effector cell functions.^20^ The basic defence functions of neutrophils contain invading microorganisms and phagocytosis of cellular debris, production of oxygen‐derived reactants, and release of protein hydrolases (Figure 1).^21^ As per the suggested mechanism, the damaged host cells raise alarm signals that activate antigen‐presenting cells (APCs).^19^ Moreover, the neutrophils, which act as a vital component of the immune response, play a significant role in the recruitment, activation and programming of APCs.^22^ These cells respond rapidly to inflammatory signals deriving from infected or injured regions outside the circulatory system. Several receptors expressed by them sense a variety of soluble inflammatory mediators ranging from cytokines to bioactive lipids, to increase the aggregation of immune cells.^23^ On a per‐cell basis, neutrophils deliver lesser cytokine molecules than macrophages and lymphocytes. However, neutrophils at the site of inflammation are usually one to two orders of magnitude higher in number than mononuclear leukocytes; therefore, they can act as an important source of cytokines, such as tumour necrosis factor (TNF), at key moments in the decision to initiate an immune response.^24^ Finally, neutrophils begin to implement a regime of microbial killing, executing programs of phagocytosis, degranulation and NETosis.^25^

The recruitment of neutrophils in most tissues in small post‐capillary venules usually follows a multi‐step pathway during inflammation: tethering, rolling, arresting, crawling and eventual transmigration (Figure 1).^26^ The neutrophil recruitment cascade is initiated by changes in the surface of endothelial cells caused by inflammatory mediators (such as histamine, leukotrienes and cytokines) delivered by leukocytes in the tissue upon encountering pathogens.^27^ Additionally, endothelial cells can be activated directly by detecting the pattern recognition receptor (PRR) of pathogens.^28^ Once activated, endothelial cells can upregulate adhesion molecules within minutes and maximize the recruitment of neutrophils through overlapping functions.^28^ Both E‐selectin and P‐selectin can capture neutrophils in circulation to bind to endothelial cells and cause neutrophils to start rolling towards the endothelial surface in the direction of blood flow. On the contrary, L‐selectin promotes secondary tethering of circulating neutrophils that are already rolling.^29^ At the site of inflammation, neutrophils perform different effector functions that lead to the clearance of invading microorganisms or promote an inflammatory response. Moreover, neutrophils are capable of producing chemotactic signals that attract monocytes and dendritic cells (DCs) and affect macrophage differentiation into predominantly pro‐ or anti‐inflammatory conditions.^30^ The rich and varied influence of neutrophils during this final stage of the inflammatory process is complex, and the exact effect, injury or repair, on tissues is not completely understood.

## 3 NEUTROPHILS MAY PROMOTE MUSCLE ATROPHY

Previous studies have shown that neutrophils account for over 90% of the circulating granulocytes and in many pathological states are usually the first cell types to invade the skeletal muscle.^31^ Frequent and severe infections in neutrophil‐deficient patients have further confirmed the importance of these immune cells in the host's defence against infection.^32^ However, muscle injury can be reduced when functionally normal neutrophils do not migrate to the injured tissues.^33^ Additionally, the pattern of leukocyte response to tissue damage shows that neutrophils infiltrate the damaged tissue and exacerbate the original damage as they assist in the removal of the damaged tissue.^34^ Thus, neutrophils can be greatly destructive and lead to severe tissue injury and can cause exacerbation of skeletal muscle damage in hours to days after damage. Neutrophil recruitment varies in number and type depending on the pathogeny in the skeletal muscle.^7^ In certain types of controlled inflammatory responses, the precise role of neutrophils is currently unclear; it includes the prevailing inflammatory conditions in skeletal muscle: sterile, nonhypoxic and nonchronic inflammatory conditions.

**FIGURE 2:**
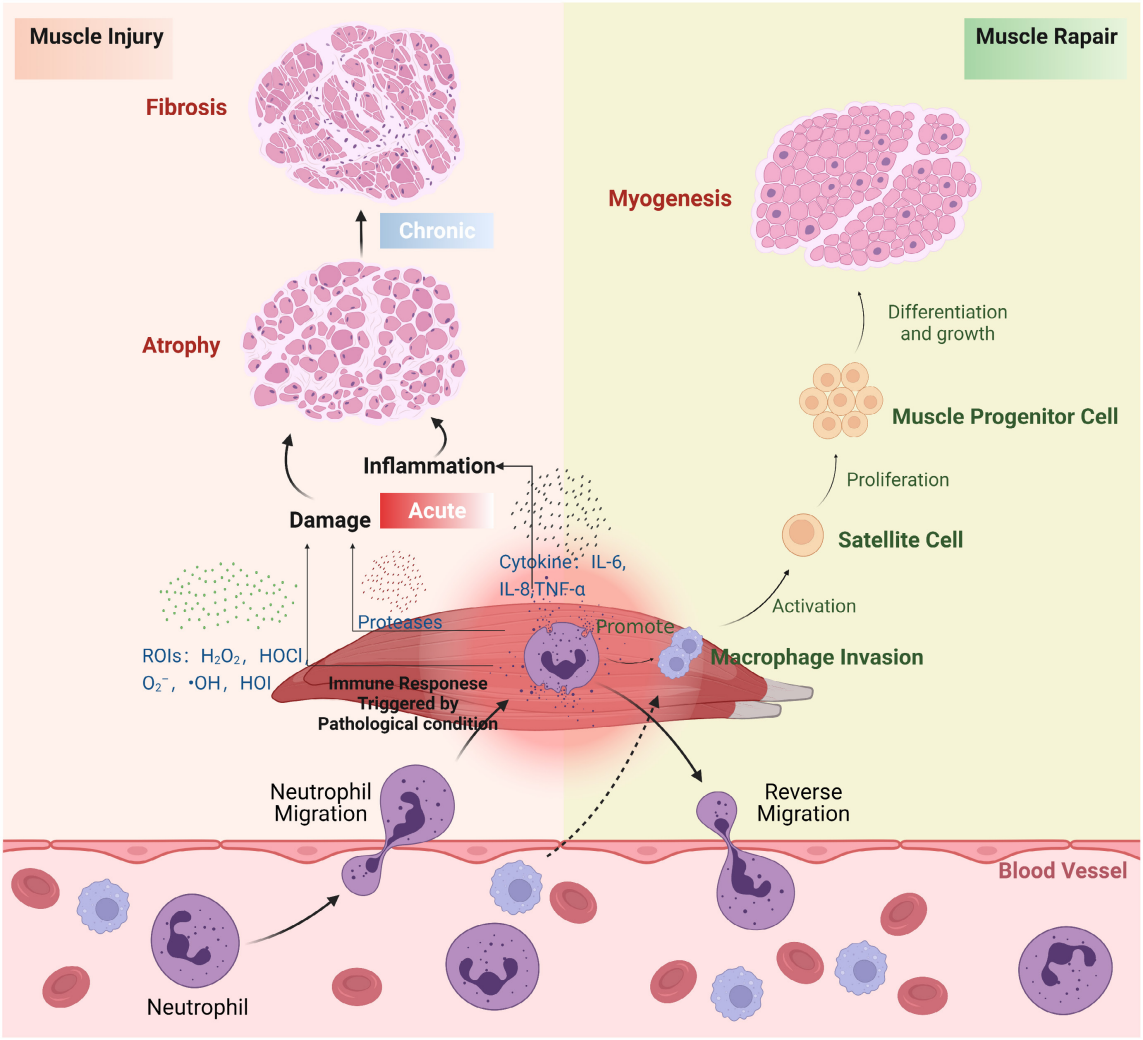
Neutrophils in muscle atrophy and repair. Neutrophils are believed to play a key role in muscle atrophy, where they can have either pro‐atrophy or pro‐repair activities. They can exert adverse effects in the degeneration phases by release proinflammatory cytokines or ROIs or proteases accelerating the muscle atrophy and fibrosis. However, macrophages could be attracted to the injured area by neutrophil signal and actively promote fibre regeneration.

In recent years, several morphological observations in vivo and in vitro suggested that neutrophils could indeed exacerbate muscle injury and atrophy through the release of free radicals and protein hydrolases.^33^, ^35^ An in vitro study of myotubular injury and tension revealed that both types of damage could lead to the release of factors that affected neutrophil chemotaxis and initiation, but only injured myotubes released factors that activated neutrophil production of free radicals.^21^ It suggests that muscle cells release inflammatory molecules that can activate neutrophils to varying degrees, and that such molecules vary with the type and intensity of the injury.^36^ Relevant scholars suggested that neutrophils impaired cultured skeletal muscle cells (myotubes) through CD18‐mediated neutrophil adhesion and production of ROS.^37^, ^38^ The skeletal muscle atrophy induced by the loading of atrophic muscle,^31^, ^39^ eccentric contractions^40^ or muscle trauma,^41^, ^42^ is associated with increased muscle neutrophil concentrations. Thus, neutrophils are considered to promote skeletal muscle injury in hours to days following damage and aggravate muscle atrophy for longer durations, mainly due to the temporal relationship between muscle neutrophil concentrations and secondary damage, and also because neutrophils can deliver potentially harmful lysosomal enzymes as well as reactive nitrogen intermediates (RNIs) and reactive oxygen intermediates (ROIs) (Figure 2).^43^, ^44^ It is established that both RNIs and ROIs exert important roles in homeostasis and inflammation.^45^ Thereinto, the successive 1‐electron reduction products of O_2_ on the way to producing water are known as ROIs. Specifically, oxygen with a reduction state that is halfway between that of oxygen (O_2_) and water (H_2_O) is referred as the term ‘intermediate’ in ROIs.^13^ As a rule, the ROIs mainly consist of hydroxyl radical (●OH), hydrogen peroxide (H_2_O_2_) and superoxide anion radical (O_2_^−^ or O_2_^●−^). Further, the ROS covers ROIs plus singlet oxygen (^1^O_2_) and ozone (O_3_).^46^ Sometimes, the terms ROS and ROIs are utilized interchangeably and may also contain hypoiodous acids (HOI) and hypochlorous (HOCI). Correspondently, the RNIs that make marked impacts on ROIs levels are nitrite (NO_2_^−^), nitric oxide radical (NO), peroxynitrite (ONOO^−^) and nitrogen dioxide radical (●NO_2_).^47^ The subset of atoms that are shared by various microbial molecules with RNIs and ROIs would generate covalent chemical reactions, and they are proven to be toxic when at high levels in immune microenvironment.^48^

Additionally, a previous study has shown that within 2 h after injury, the concentration of neutrophils in muscle increased and remained for at least 48 h above the normal concentrations.^31^, ^39^, ^42^ During this time, the muscles would degenerate further (secondary damage).^41^ Moreover, one interesting study indicated that neutrophils could extremely amplify muscle injury led by the use of modified muscles, accelerating muscle atrophy and fibrosis.^49^ The addition of myeloperoxidase (MPO)‐rich neutrophils to muscle with increased load in the presence of elevated superoxide dismutase (SOD) activity and reduced peroxidase activity will promote sarcolemma lysis.^50^ MPO by itself is not lethal but can effectively enhance the lethality of ROIs; lack of MPO or addition of MPO inhibitors can reduce the lethality of neutrophils.^51^ In non‐stressed neutrophils, MPO is stored in islamophilic granules. Once the neutrophils are bound, however, the enzyme is delivered into the phagocytic vesicles or extracellular space.^18^ Also, in MPO knockout mice, membrane lysis of flounder muscle after exercise was significantly reduced by 52% compared to wild‐type mice,^49^ demonstrating that MPO‐containing neutrophils and their activators, such as proinflammatory cytokines, promote muscle injury and atrophy.

In addition, infiltrating neutrophils can release chemical signals to attract phagocytes, such as macrophages and monocytes, to the damaged region.^52^ ROS originating from these phagocytes increases gene expression of inflammatory cytokines in the skeletal muscle which results in increased aggregation of phagocytes to the injured muscle tissues.^7^, ^53^, ^54^ Many inflammatory cells release inflammatory cytokines (e.g. interleukin [IL]‐6, IL‐1 and TNF‐α) and ROS, and this microenvironment may be a key driver of the downstream effects of inflammation and myofibrillar lysis.^7^ These findings are novel and provide evidence for the mechanism by which neutrophils can modulate inflammation by inducing macrophage infiltration, leading to muscle atrophy in the long term.

Neutrophil infiltration have been shown to occur in the following exercised‐injured muscles: plantaris,^55^ rectus femoris,^56^ soleus,^57^ tibialis anterior,^58^ white vastus^57^ and vastus lateralis.^59^ Among different muscle types, however, the concentration of macrophages or neutrophils appears to be vary in motorial and sedentary control muscles.^57^, ^60^ These findings may be related to those areas of the muscle that are dominated by fast fibres. In the hindlimb suspension (HS) model, significant increases in myoneutrophil and macrophage concentrations occurred during exacerbation of ultrastructural signs of injury and fibrous necrosis, indirectly supporting the possibility that invasive inflammatory cells are involved in muscle injury.^61^, ^62^ McArdle et al.^63^ informed that muscle contraction caused no significant damage and increased the production of muscle‐derived superoxide anions. The superoxide anion and the downstream reaction product, hydrogen peroxide, have been reported to cause oxidative modification of plasma proteins which led to neutrophil chemotaxis^64^ and enhanced neutrophil activation (i.e. production and degranulation of ROS).^65^ In‐depth research demonstrated that oxidation products and hypochlorite produced by neutrophils mediated phagocytosis and destruction by neutrophils.^66^ Additionally, inhibiting neutrophil adhesion and ROIs production with anti‐CD18 and intracellular adhesion molecule‐1 antibodies has been reported to attenuate neutrophil‐mediated damage to endothelial cells and myocardial myogenic cells.^67^, ^68^ Moreover, CD18 knockout mice revealed a more direct link between neutrophil infiltration and muscle atrophy.^50^

In response to fibre injury, restorative progenitor cell activation competes with the ongoing fibrotic process and ultimately inhibits the recovery of atrophic muscle function. Repeated cycles of injury, repair and fibrosis can result in satellite cell depletion and fibre degeneration.^70^ Therefore, neutrophils, one of the main cellular components of microbial destruction in the human body, also play a vital role in muscle atrophy.

## 4 DO NEUTROPHILS PARTICIPATE IN MUSCLE REPAIR?

To date, the role of neutrophils in muscle atrophy has not been elucidated for several pathological conditions. Some scholars showed that the aggregation of neutrophils and macrophages was not consistent with the rapid power reduction seen 2 h after reloading in an HS model. This observation supported the assumption that the original mechanism by which reloading leads to damage is a mechanically modulated activity in which neutrophils play a minor role.^70^ Thus, from the studies in the HS model, it was concluded that: (1) an increase in neutrophil concentration was not associated with any decrease in muscle strength and (2) the inability to activate the contractile machinery was the primary mechanism for the loss of early strength production after reloading.^71^ These results suggested that neutrophil infiltration is highly regulated and is effectively eliminated during modified mechanical loading, and that no significant damage is caused to myofibres unless the activation state of neutrophils is altered by the presence of lipopolysaccharide (LPS).^17^ Meanwhile, previous studies have demonstrated that despite the presence of large numbers of neutrophils, reloading after hindlimb unloading caused minimal damage to muscle fibres (1%–2%).^49^ Additionally, functional and immunohistological analyses in vitro also revealed that the loss of muscle strength linked to hindlimb unloading and reloading did not correlate with the time course of neutrophil aggregation.^71^ In reality, the muscle atrophy and recovery modulated by physiological processes in unloaded and reloaded muscles are distinct from chemical and ischaemic damage or muscle crush, and they could accordingly result in different neutrophil responses. In the former case, there is essentially no muscle damage, and the goal is maximum muscle recovery, while the latter requires myofibrillar breakdown, phagocytosis, satellite cell proliferation, myotube formation and eventually tissue remodelling to re‐establish homeostasis in vivo. The mechanism of neutrophil infiltration is unclear, but the resulting sustained inflammatory injury is thought to be an important factor in initiating the repair process.^17^

Some preliminary results have demonstrated that inhibiting the infiltration of neutrophils impairs regenerative response in muscles while having little influence on non‐muscle cellular proliferation.^72^ By clearing tissue fragments from damaged areas through phagocytosis^41^ and activating satellite cells, alternatively, neutrophils could promote muscle regeneration.^73^, ^74^ In an elaborate study of a crush‐injury model, TNF‐α was initially released by mast cell degranulation, after which neutrophils started accumulating in the muscle bed and released more TNF‐α, and this was followed by macrophage infiltration.^75^, ^76^ Resident macrophages surrounding the epimysium and perimysium of the whole muscle and fascicles, respectively, also play a key role in the early stages of various acute muscle injuries by producing chemokines that attract neutrophils from the bone marrow that migrate outwards into the tissues.^77^ Moreover, infiltrated neutrophils can further promote macrophage inflammatory secretion and result in macrophage phenotype conversion, possibly activate satellite cells, and accelerate muscle repair (Figure 2).^78^ The study showed that low‐density lipoproteins (LDLs) together with CD68 could reinforce the inflammatory secretion and phagocytosis by macrophages^79^, ^80^; during this stage, particularly, the release of MPO from neutrophils promoted these bindings by oxidative modification for LDLs^81^ and these interactions may exert a crucial role in muscle repair. Similarly, MPO was also an important ligand for CD206,^82^ which could regulate the macrophage functions by reducing muscular injury and inflammation.^83^ More importantly, it is well‐known that the transition between macrophage phenotypes is essential for muscle regeneration and neutrophils seem vital for the phenotype shift of macrophages from M1 to M2 during muscle repair.^84^ Several experimental evidences strongly suggested that the time course of neutrophil apoptosis coincides with macrophage phenotypes shift in the HS model of muscle injury and repair.^84^ Further, the increased expression of TGF‐β also represented the shift towards the M2 phenotype by phagocytizing apoptotic neutrophils.^85^ As previous research reported, within a few days of muscle toxin‐induced injury, macrophages and immature dendritic cells became the main immune cell type at the site of injury, aiding tissue repair.^86^ Arnold L et al., as well, supported the fact that necrotic fibres are phagocytosed by macrophages in these models. Then, the macrophages energetically promote re‐myogenesis via the impacts on satellite cells.^87^ Additionally, human satellite cells in vitro were proven to directly attract macrophages by releasing factors release,^88^ which may provide further help during muscle repair. To date, despite various immune cells invasion after muscle injury,^89^, ^90^, ^91^ only neutrophils and macrophages are reported to be conducive to muscle regeneration. These findings suggest that the neutrophils together with macrophages promote muscle differentiation and growth after muscle injury.

## 5 CONCLUSIONS

Several myopathies, mainly due to their muscle atrophy and fibrosis events, involve an immune cellular inflammatory response which probably influences the outcome. Throughout this review, we have presented both physiological and pathological findings that reveal a close connection between neutrophils and muscle atrophy. Despite some encouraging findings as evident from changes in neutrophil numbers or effects in atrophied muscles in the examples that have been highlighted, challenges and controversies in the field remain. There remains a large knowledge gap between our current understanding of specific molecular mechanisms that mediate muscle atrophy and fibrosis, and the influence of neutrophil infiltration in atrophied muscles. Furthermore, even with in‐depth knowledge, achieving accurate targeting and appropriate intervention for neutrophils in atrophied muscles will certainly be a major challenge while developing effective treatments. Given the damage caused by neutrophils in atrophied muscles and the prominent role of neutrophils in muscle repair, the interaction between neutrophils and muscle needs to be improved understood.

## AUTHOR CONTRIBUTIONS

**Zongqi You:** Writing – original draft (equal); writing – review and editing (equal). **Xinying Huang:** Software (supporting); validation (supporting). **Yaoxian Xiang:** Resources (supporting); supervision (supporting). **Junxi Dai:** Visualization (supporting). **Junjian Jiang:** Project administration (lead). **Jianguang Xu:** Project administration (equal).

## CONFLICT OF INTEREST

The authors confirm that there are no conflicts of interest.

## Data Availability

The data that support the findings of this study are available from the corresponding author upon reasonable request.
